# GRIP-Lung: Generative Model of Response to Drug-Induced Perturbation in Lung Cancer

**DOI:** 10.3390/ijms27073264

**Published:** 2026-04-03

**Authors:** Zhijin Fu, Yanjiao Li, Zhenshun Du, Denan Zhang, Lei Liu, Qing Jin, Xiujie Chen, Hongbo Xie

**Affiliations:** Department of Pharmacogenomics, College of Bioinformatics Science and Technology, Harbin Medical University, Harbin 150086, China; 19817962167@163.com (Z.F.); 2023020543@hrbmu.edu.cn (Y.L.); 2023020615@hrbmu.edu.cn (Z.D.); zhangdenan@ems.hrbmu.edu.cn (D.Z.); liulei@ems.hrbmu.edu.cn (L.L.); jinqing@hrbmu.edu.cn (Q.J.)

**Keywords:** lung cancer, generative adversarial network, drug response

## Abstract

The prediction of drug response would significantly improve the treatment of lung cancer. Tumor heterogeneity and complex signal transduction pathways lead to varied treatment effects among patients, but traditional computational approaches struggle to model the nonlinear, high-dimensional relationship between genes and drug responses. In order to develop a Generative Adversarial Network (GAN)-based model that can predict drug-induced gene expression profiles from lung cancer cell lines, we developed GRIP-Lung (Generative Model of Response to Drug-Induced Perturbation in Lung Cancer). By making use of biologically informed embeddings of cell line identity as well as drug treatment conditions, this model is able to gain a fairly good understanding of cell types and their transcriptional perturbations induced by different drugs. The GRIP-Lung model displayed reasonably good prediction ability in terms of predictive accuracy and showed high concordance between the predicted and experimental expression profiles. We not only predicted transcriptional changes induced by drug therapy but also used single-sample Gene Set Enrichment Analysis (ssGSEA) to classify post-treatment response states based on characteristic molecular biomarkers, offering a means for selecting effective drugs to target specific heterogeneity within lung tumors. The proposed GRIP-Lung framework faithfully reproduces drug-induced transcriptional perturbations in lung cell line models. By integrating biologically informed embeddings and adversarial learning, the model advances drug response prediction. This makes it a flexible computational tool for drug repositioning.

## 1. Introduction

Understanding drug response is essential for the study of cancer treatment because determining the efficacy of personalized therapy directly is crucial. Evaluating drug responses allows clinicians to distinguish which kinds of drug treatment regimens will be more advantageous for a particular patient, and through this approach, they can better tailor drug selection, dosages, and combination regimens to enhance a patient’s therapeutic response [1,2]. Studies on drug responses have elucidated the mechanisms underlying treatment resistance in cancer, thus providing a scientific basis for the development of novel anti-resistance therapies and advancing the implementation of precision medicine in oncology [3].

Although tremendous achievements have been made in research on drug responses, much remains unknown, such as the interindividual differences leading to varied therapeutic outcomes [4,5,6]. Additionally, the processing and interpretation of large-scale multi-omics data (e.g., genomics, transcriptomics, and proteomics) to extract clinically relevant information remain major challenges [7]. To overcome these difficulties, recent studies have focused on how to integrate large-scale datasets to characterize cell heterogeneity and drug responses using integration methods. For example, Hao et al. assembled an extensive single-cell dataset by integrating data from all publicly accessible single-cell resources, aiming to develop a foundation model, scFoundation, designed to decode the “language” of cells. The decoder of scFoundation generated gene-level contextual embeddings and demonstrated strong performance across various tasks, including read-depth enhancement, drug response and sensitivity prediction at the single-cell level, perturbation modeling, and cell type annotation [8]. While foundation models such as scFoundation offer computational insights into cellular behavior, experimental studies aim to directly associate single-cell transcriptomes with drug responses. By integrating single-cell transcriptomics with high-throughput drug screening in high-grade serous ovarian cancer, Dini et al. applied a 96-plex scRNA-seq approach to analyze the responses of 36,000 cells to 45 drugs, representing 13 mechanisms of action [9].

Predicting changes in gene expression levels upon drug perturbation is important for revealing the biological mechanisms underlying different responses to drugs, including their sensitivity, resistance, and side-effects [10,11,12]. In contrast to static transcriptome profiling, which reflects only the final state of drug response, predictive models help in understanding the dynamic regulation leading to the establishment of drug response phenotypes. By predicting changes in specific pathways and genes after treatment with drugs, early-stage modifications that impact the fate of various therapeutic responses can be identified. Accordingly, accurately predicting drug outcomes has the capacity to improve knowledge of heterogeneity in drug responses and promote the development of precision oncology.

Deep learning based on neural network architectures can combine multiple omics datasets and process vast and complex data to discover detailed patterns and associations that have been difficult to find with ordinary approaches [13,14,15]. Accordingly, deep learning is capable of dissecting drug responses, particularly in predicting outputs from drug response models. Moreover, the use of these methods provides a more comprehensive understanding of drug responses. Through many studies, deep learning has made significant contributions to the large-scale data mining of drug responses. For example, Zheng et al. developed a computational framework, UNAGI, to model the temporal dynamics of cellular behavior during complex disease progression. This tool enables a detailed analysis of gene regulatory networks and cellular transitions, offering insights into potential therapeutic targets and drug candidates for idiopathic pulmonary fibrosis. By simulating compound responses and disease trajectories, UNAGI facilitates drug repositioning and mechanistic investigation [16].

Lung cancer causes approximately 20% of all cancer deaths and continues to be the main cause of death from cancer worldwide [17,18]; its advanced heterogeneity has led to many problems that have prevented the identification of effective therapeutic options. Lung adenocarcinoma (LUAD) represents the most prevalent histological subtype of lung cancer and continues to impose a substantial global health burden. Despite advances in targeted therapy and immunotherapy, recent clinical studies have highlighted the persistent heterogeneity in treatment response and prognosis among patients. For instance, contemporary investigations evaluating pembrolizumab-based combination strategies have demonstrated variable therapeutic benefits depending on molecular and clinical characteristics, underscoring the complexity of precision treatment for LUAD [19]. In parallel, emerging biomarker studies have explored novel molecular indicators for prognosis and immunotherapy responses, yet robust and universally applicable predictive markers remain limited [20]. These findings collectively emphasize that the therapeutic response in lung cancer patients is highly individualized and difficult to predict using conventional clinical parameters alone. The reliable prediction of patient-specific responses is crucial for guiding individualized treatments. One reason for this is that compared with most other cancers, lung cancer has a relatively large number of public databases that contain detailed gene expression profiles as well as annotated drug responses, which makes it the perfect field to explore using deep learning models.

In this study, we present a Generative Adversarial Network (GAN) framework, GRIP-Lung (Generative Model of Response to Drug-Induced Perturbation in Lung Cancer), for predicting drug responses in lung cancer, optimized through a systematic evaluation of various generator and discriminator architectures. By applying this model to lung cancer cell gene expression data, we predict drug response profiles following treatment. The model accurately captures gene expression changes induced by drug perturbation and demonstrates good robustness in inferring biological functions at the cellular level. Furthermore, the model is flexible enough to dissect drug-induced perturbations across gene expression in bulk patient RNA-sequencing datasets. This capability enables the identification of key genes that are crucial for drug responses in lung cancer, providing valuable insights into the molecular mechanisms underlying treatment efficacy. Building on this capability, we define six drug response states using ssGSEA to analyze key biomarker transcriptomes for each post-treatment state. We then present a framework that can differentiate among a wide range of drug responses based on RNA-seq data. Integrating GAN-based drug response prediction with gene expression information could help improve personalized cancer therapy and inform treatment strategies for lung cancer. The package and source code are available on GitHub at https://github.com/XieHB-lab/GRIP-Lung (accessed on 1 April 2026).

## 2. Results

### 2.1. Overview of the GRIP-Lung Framework and Performance Evaluation

**Figure 1:**
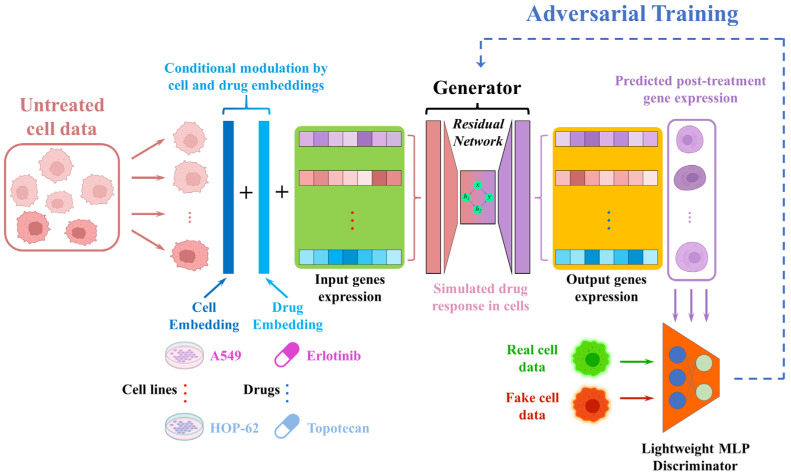
Workflow of the GRIP-Lung framework. Pre-treatment gene expression profiles are combined with learnable cell and drug embeddings to incorporate condition-specific information. The resulting representation is passed to a multilayer residual network generator designed to model drug-induced transcriptional perturbations and outputs predicted post-treatment expression profiles. Adversarial training further enhances biological plausibility, with a lightweight discriminator distinguishing real from generated profiles while both networks are optimized jointly.

GRIP-Lung is a generative model specifically formulated for predicting the drug-induced gene expression profile of lung cancer cell lines. Unlike conventional generative models, GRIP-Lung embeds cell lines and drug treatment conditions in the input representation to generate sample-specific perturbation patterns, thus capturing drug-induced perturbation patterns specific to individual samples. The workflow of GRIP-Lung is detailed in Figure 1.

Currently, only the use of lung cancer cell lines is allowed, where each cell line and drug are encoded by learnable embedding vectors, and their values are directly added element-wise into the normalized baseline gene expression profiles of their cell lines to generate a context-enhanced input. This representation passes through a multilayer residual network, which captures complex nonlinear dependencies between genes. GRIP-Lung has been trained only on lung cancer cell lines; it is free of organ-specific priors and thus can predict drug-induced transcriptional perturbations by considering only the transcriptome of the cell line and drug properties. This gives GRIP-Lung the potential to correctly predict the gene expression signatures of different drugs while maintaining biological fidelity. GRIP-Lung thus provides a framework to model drug-specific transcriptional responses on a cell type-specific basis.

**Figure 2:**
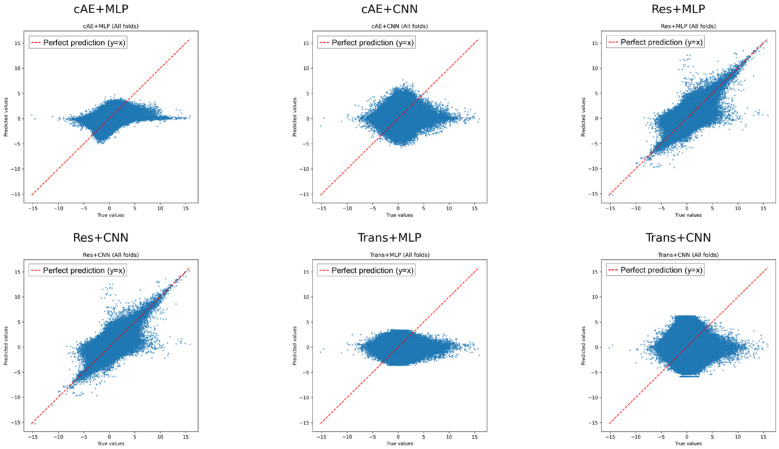
Correlation between predicted and actual gene expression profiles on the test set for six generator–discriminator combinations under 5-fold cross-validation. The Residual–MLP model exhibited the highest correlation, indicating superior generalization performance across known cell lines. Scatter plots showing predicted versus true gene expression values on the test set for six generator–discriminator combinations under five-fold cross-validation. Each point represents a gene expression value for a given cell line and drug condition. The Residual–MLP model exhibits the strongest alignment with the diagonal y = x line, indicating the highest correlation and best generalization performance across known cell lines, while other combinations show varying degrees of deviation. Overall, the predicted values from the non-residual and CNN-based generators show lower concordance, reflecting reduced predictive accuracy compared with the Residual–MLP model.

To investigate generalization across known cell lines, we performed a five-fold cross-validation study using all six combinations of generators and discriminators. The prediction truth correlations of each model on the test set are shown in Figure 2; the Residual–MLP model achieved the highest correlation.

**Figure 3:**
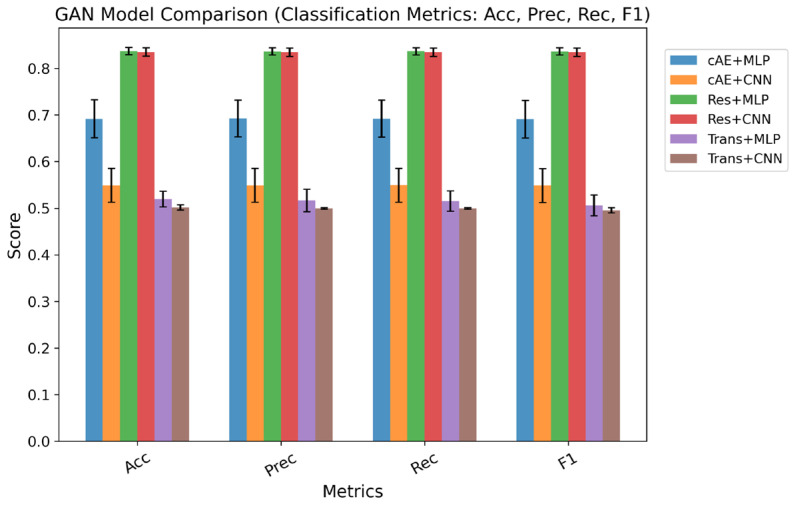
Performance comparison of different model combinations in terms of accuracy, precision, recall, and F1-score. The bar plot shows the evaluation metrics accuracy (Acc), precision (Prec), recall (Rec), and F1-score for each model combination under five-fold cross-validation. Each color represents a different generator–discriminator pair: cAE + MLP, cAE + CNN, Res + MLP, Res + CNN, Trans + MLP, and Trans + CNN. The Residual–MLP model (Res + MLP) consistently achieves the highest scores across all four metrics, indicating strong and balanced predictive performance, whereas non-residual and CNN-based generators show lower or less consistent performance. Error bars represent the standard deviation across cross-validation folds.

Model performance was assessed using the following indices: MSE, coefficient of determination (R^2^), accuracy (ACC), precision (Prec), recall (Rec), and F1 score. As summarized in Table 1 and Figure 3, the Residual–MLP architecture consistently outperformed the other models across most evaluation metrics; it had the lowest MSE (0.337) and the highest R^2^ (0.652), thus indicating that it achieved better regression performance. Moreover, in the corresponding classification task that was obtained by applying a threshold to the regression output, continuous gene expression predictions were binarized using a zero threshold after standardization, where values greater than 0 were considered upregulation and values less than or equal to 0 were considered downregulation, allowing the computation of classification-based metrics. A prediction was considered correct when the direction of regulation (upregulation or downregulation) was consistent between the predicted and true post-treatment expression values. The Residual–MLP model also demonstrated strong and balanced performance, with ACC, Prec, Rec, and F1 score all being approximately 0.84. Since it performed well in both the regression and classification tasks, the Residual–MLP model was selected as the optimal solution. Its low prediction error indicates high prediction accuracy, and its consistent results across different models and evaluation metrics show that it effectively captures the underlying patterns between genes and drugs. In addition, its stable performance across multiple metrics suggests that the model is robust and may reflect meaningful biological relationships.

To further evaluate whether the residual conditional GAN architecture provides advantages over simpler modeling strategies, we compared GRIP-Lung with two commonly used baselines: Linear Regression and a standard autoencoder. Linear Regression was performed to assess whether drug-induced transcriptional changes could be captured by purely linear mapping from baseline to post-treatment expression. The autoencoder baseline was trained to reconstruct post-treatment profiles from baseline inputs without adversarial learning or residual connections. GRIP-Lung achieved the best predictive performance (R^2^ = 0.629, MSE = 0.343), outperforming both Linear Regression (R^2^ = 0.547, MSE = 0.416) and the standard autoencoder (R^2^ = 0.322, MSE = 0.633). A detailed comparison is provided in Figure S1. These results suggest that incorporating residual learning and adversarial optimization improves the modeling of transcriptional perturbations beyond linear or reconstruction-based approaches.

### 2.2. Validation of GRIP-Lung on Erlotinib-Treated Samples

Erlotinib is a frequently used EGFR tyrosine kinase inhibitor that is indicated for the treatment of non-small cell lung cancer (NSCLC), especially in NSCLC patients with activating EGFR mutations.

**Figure 4:**
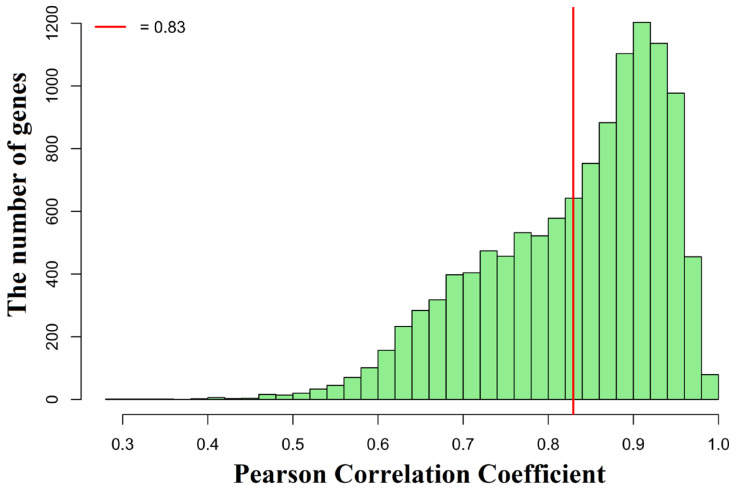
Distribution of gene-wise Pearson correlation coefficients between predicted and actual gene expression values. The histogram shows the number of genes (*y*-axis) corresponding to different Pearson correlation coefficients (*x*-axis) calculated between predicted and experimental gene expression values for multiple erlotinib-treated lung cancer cell lines. Each gene’s correlation coefficient was computed by pairing the predicted expression value with the observed experimental value across all samples. Most genes exhibit strong correlations, primarily ranging from 0.6 to 1, indicating that the model accurately captures gene-level expression patterns. The vertical red line represents the median Pearson correlation coefficient of 0.83, highlighting that at least half of the genes show high agreement between predicted and observed expression.

To better assess the accuracy and biological relevance of GRIP-Lung, we used erlotinib as a typical example. Using the GAN model, we produced erlotinib-treated gene expression profiles for multiple lung cancer cell lines and compared them with the experimental expression profile data. The Pearson correlation coefficient was calculated by computing the corresponding expression value pairs between each predicted and observed sample value for every gene. As illustrated in Figure 4, most genes showed high degrees of agreement, with correlation coefficients primarily ranging from 0.6 to 1, indicating that the majority of genes had expression patterns similar to those predicted by the model.

**Figure 5:**
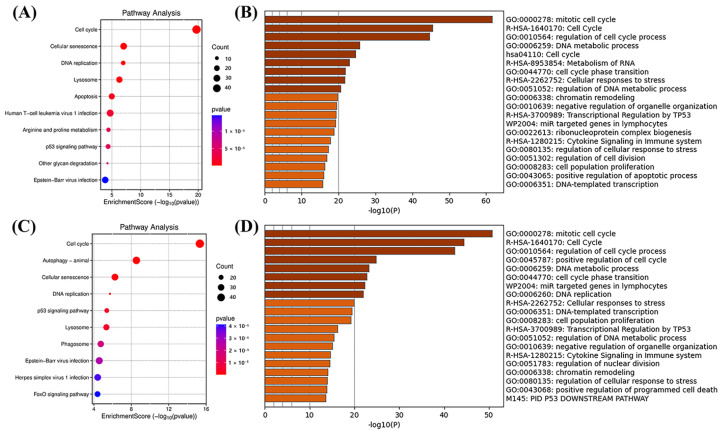
Enrichment analysis of DEGs between the predicted and experimental groups. DEGs were identified by comparing predicted or experimental gene expression profiles of erlotinib-treated versus untreated lung cancer cell lines. KEGG and Gene Ontology (GO) enrichment analyses were performed to assess the biological pathways and processes associated with these DEGs. (**A**) KEGG enrichment of GAN-predicted DEGs after erlotinib treatment. (**B**) GO enrichment of GAN-predicted DEGs after erlotinib treatment. (**C**) KEGG enrichment of actual DEGs after erlotinib treatment. (**D**) GO enrichment of actual DEGs after erlotinib treatment. Both predicted and experimental DEGs show consistent enrichment in cell-cycle-related pathways, cellular senescence, and p53 signaling pathways, reflecting the known mechanism of erlotinib in inhibiting EGFR signaling, inducing G1/S cell-cycle arrest, and suppressing proliferation. The comparison illustrates that the model reliably captures the key biological effects of erlotinib treatment.

In addition, by comparing model-predicted and experimentally measured erlotinib-treated vs. untreated samples to generate differentially expressed genes (DEGs), we identified DEGs that showed similar patterns in both types of samples. KEGG pathway enrichment analysis also revealed a consistent set of bioprocesses from the predicted and experimentally derived DEGs, such as cell-cycle processes downstream of EGFR inhibition. Furthermore, both the predicted and experimentally observed DEGs were involved mainly in cellular senescence and p53 signaling pathways; a comparison of the results of the GO enrichment analysis supported this conclusion. Both the predicted and experimentally determined DEGs were also enriched in cell-cycle-related pathways. This result corresponds with the known mechanism of action of erlotinib, which indirectly regulates cell-cycle progression, leading to G1/S cell-cycle arrest, the inhibition of proliferation, and the induction of apoptosis [21,22,23]. Therefore, the cell-cycle-related results obtained from the RNA-seq expression profile are consistent with those reported in clinical studies. The enrichment of DEGs between the predicted and experimental groups is shown in Figure 5.

Among the top predicted differentially expressed genes (DEGs) in erlotinib-treated lung cancer cell lines, *DDIT3* (also known as *CHOP*), *CHAC1*, and *ATF3* were upregulated, and all of these genes are downstream targets of EGFR signaling [24,25,26]. Additionally, in certain cell lines, the model predicted increased *HSPA6* expression, which is associated with acquired resistance to EGFR inhibition [27]. The lists of predicted DEGs and of those both predicted and experimentally validated are shown in Supplementary Table S1. These findings demonstrate the robust ability of the model to correctly capture gene expression changes elicited by treatment with erlotinib that closely reflect known resistance-associated markers and pathways. This feature serves to validate the model and thereby augment the value of this tool in dissecting drug mechanisms and stratifying responses in preclinical models.

### 2.3. Transcriptional Responses of A549 Cells to Multiple Drug Treatments

To determine whether our model can separate drug-specific transcriptional profiles within a single cell line, we selected the A549 cell line, which has a KRAS mutation and wild-type EGFR [28].

**Figure 6:**
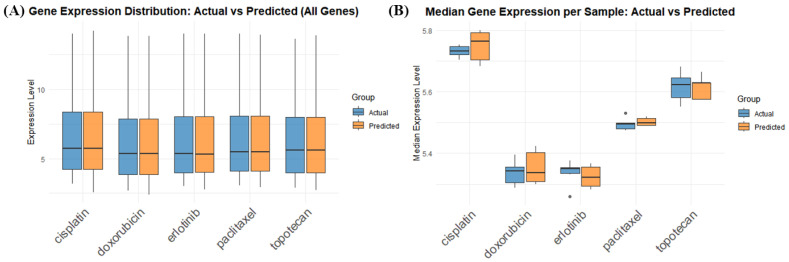
Comparison of gene expression distributions between predicted and experimental profiles for A549 cells treated with different drugs. Box plots were used to compare predicted and experimentally measured gene expression values. (**A**) Distribution of expression levels for all genes after treatment with five drugs (cisplatin, doxorubicin, erlotinib, paclitaxel, and topotecan). The blue and orange boxes represent the actual and predicted expression values, respectively. The plot shows that the overall gene expression levels remain stable across all drugs, with similar distributions between predicted and experimental profiles, indicating that the model effectively captures the global gene expression patterns. (**B**) Median gene expression per sample for each drug. The box plots display the median expression levels across all genes in each sample, illustrating that predicted median values closely match experimental values. While overall medians remain in the range of approximately 5.3–5.8, differences between drugs are observed, reflecting drug-specific gene expression effects.

We used box plots to compare the predicted and actual gene expression profiles of A549 cells treated with each drug, illustrating both the overall distribution of gene expression values and the median expression level for each sample. As shown in Figure 6, the total gene expression remained stable, and the distribution and median values across all genes were highly similar between the predicted and experimental profiles.

The predicted expression profiles revealed distinct gene regulatory signatures for each treatment. We performed differential gene expression and enrichment analyses between the predicted gene expression profile of the A549 cell line after drug treatment and that of the untreated control and compared the results with the corresponding actual values. Despite the involvement of multiple drugs, the predicted results remained consistent with the actual values in terms of biological function. Pathway enrichment analysis of the predicted gene expression changes further supported drug-specific effects. For instance, DEGs associated with the cell cycle were still identified in the predicted results for paclitaxel-treated A549 cells (Figure S2). Paclitaxel is a microtubule-stabilizing agent that binds β-tubulin, suppresses microtubule dynamics, and induces G2/M arrest by disrupting spindle assembly and chromosome segregation, thereby strongly affecting cell-cycle regulation [29,30].

### 2.4. Patient-Level Validation of GRIP-Lung on Clinical Transcriptome Data

To provide preliminary patient-level validation, we applied GRIP-Lung to bulk RNA-seq data derived from lung cancer patients (GSE165019). Paired gene expression data before and after erlotinib treatment were available for four patients (Patient 2 and Patients 4–6). To bridge the domain gap between patient samples and in vitro cell lines, each patient transcriptome was mapped to its most transcriptionally similar lung cancer cell line on the basis of baseline gene expression similarity. The corresponding cell line embedding was then used as a proxy for the patient’s molecular context during prediction.

**Figure 7:**
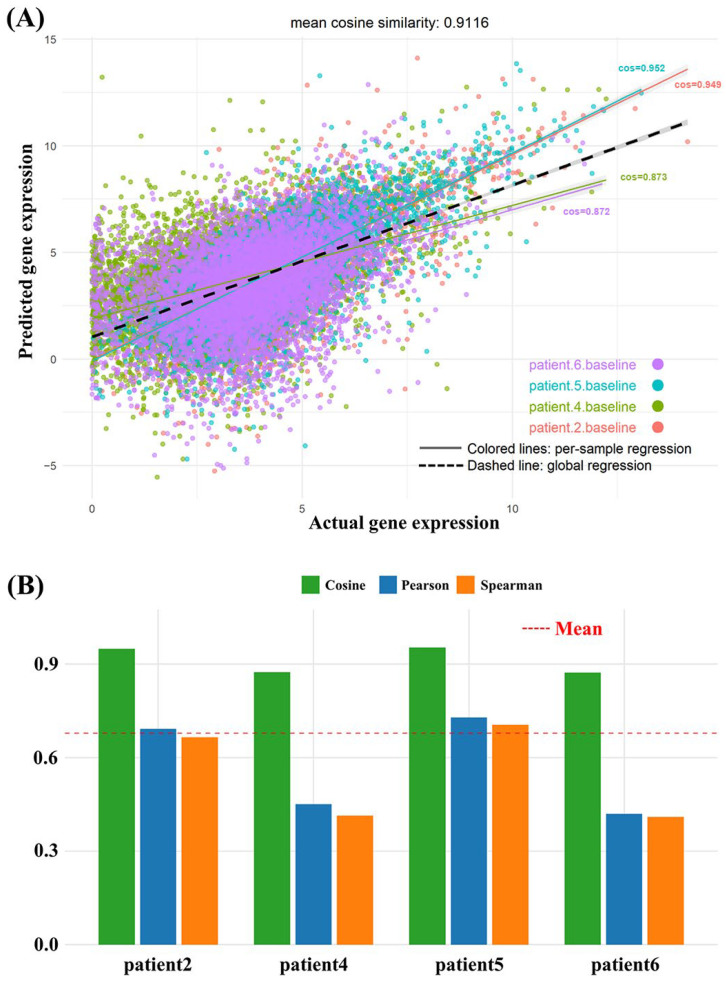
Assessment of GRIP-lung prediction accuracy on patient gene expression profiles. (**A**) Concordance between predicted and actual post-treatment gene expression profiles for patient samples. (**B**) Similarity metrics based on cosine similarity, Pearson correlation, and Spearman correlation comparing predicted and actual gene expression profiles.

GRIP-Lung can predict post-treatment gene expressions and determine therapeutic drug-induced transcriptional changes. As shown in Figure 7A, although those samples were not used for modeling, the predicted gene expression profiles were in good agreement with the real gene expression data. Similarity analyses through cosine similarity (Figure 7B), the Pearson correlation coefficient, and the Spearman correlation coefficient revealed very high consistency between the predicted results and the real results, suggesting that the model accurately fits real patient-derived transcriptomes and can be adaptively used for distinct tumors. GRIP-Lung can be remarkably useful in precision oncology, especially for forewarning the potential occurrence of therapeutic resistance and offering effective tumor-specific treatment. To further evaluate the magnitude accuracy of the predictions, we additionally calculated RMSE and MAE specifically for the DEGs identified from the true post-treatment profiles versus the baseline profiles. The identified DEGs are listed in Supplementary Table S2. As summarized in Supplementary Table S3, GRIP-Lung maintained relatively low RMSE and MAE values across patients, supporting its ability to capture not only directional trends but also the magnitude of transcriptional changes.

To assess whether GRIP-Lung truly models patient-specific responses rather than simply transferring perturbations from similar cell lines, we implemented a “nearest neighbor perturbation” baseline, in which the drug-induced Δ expression from the most similar cell line was directly added to the patient’s baseline profile. Genes yielding biologically implausible negative predicted values were excluded prior to evaluation. Compared with this baseline approach (1.315), GRIP-Lung achieved a lower RMSE (1.0957), indicating superior predictive accuracy beyond simple linear perturbation transfer. Detailed comparisons are provided in Figure S3. To further examine the robustness of this comparison and the sensitivity to the cell line–patient matching strategy, we repeated the analysis using alternative pairings in which patient samples were matched with non-corresponding lung cancer cell lines. Under these conditions, gene-level correlations were consistently reduced, further supporting the importance of biologically informed matching.

To further assess whether the observed predictive performance could have arisen from random associations, we performed a permutation test by randomly shuffling gene expression labels and recalculating the prediction error. Compared with the observed data, the permuted data produced substantially greater errors, indicating that the model captures meaningful transcriptional relationships rather than random patterns (Figure S4).

### 2.5. Drug Post-Treatment Response State Score

**Figure 8:**
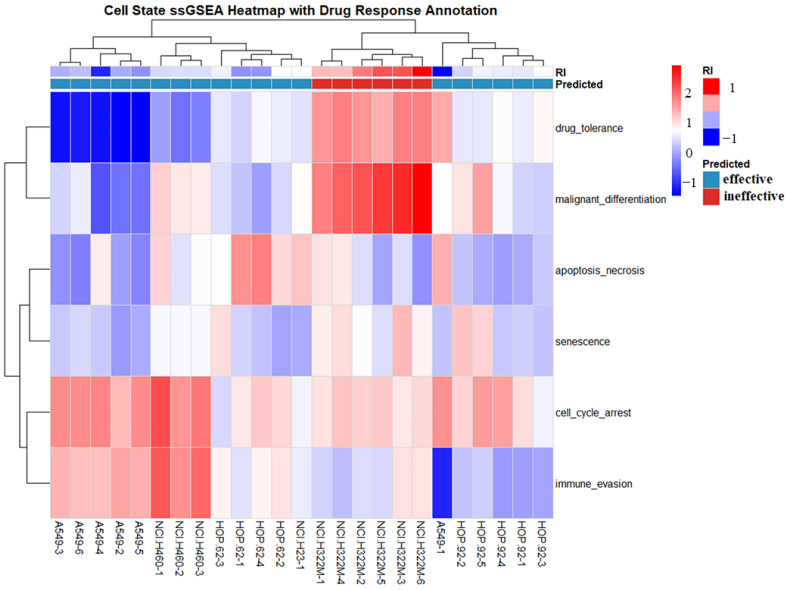
Deciphering drug response through single-sample gene set enrichment analysis (ssGSEA) of transcriptomic states. The heatmap illustrates the enrichment scores of six predefined biological states across various cell line samples treated with bortezomib. Rows represent the specific biological states, while columns represent individual cell line samples (e.g., A549, NCI-H460, HOP-62, etc.). Color intensity in the heatmap reflects the ssGSEA scores, where red indicates higher enrichment (activation) and blue indicates lower enrichment (suppression) of the respective pathway. The “RI” (resistance index) top bar shows the calculated index for each sample, with red representing RI > 0 and blue representing RI ≤ 0. The “Predicted” top bar categorizes samples into “effective” or “ineffective” based on the RI values, demonstrating the model’s ability to classify drug sensitivity based on transcriptomic signatures. Dendrograms on the top and left represent unsupervised hierarchical clustering of samples and biological states, respectively.

Transcriptional programs related to apoptosis, ferroptosis, sustained cell-cycle arrest, or senescence were operationally categorized as potentially effective responses, as they reflect stress-associated growth suppression in cancer cells under acute in vitro drug exposure conditions. In this framework, the drug-induced upregulation of these gene programs was interpreted as indicating treatment-associated cytotoxic or antiproliferative effects. We acknowledge that certain biological processes, such as autophagy and senescence, are context-dependent and may have pro-survival or pro-tumorigenic effects in different biological settings. In contrast, transcriptional signatures primarily associated with drug resistance, malignant progression, or immune escape were categorized as ineffective responses and were unlikely to confer therapeutic benefits under the same experimental conditions. We used the logGI_50_ data from the NCI-60 cell line panel to define drug sensitivity. A drug was considered effective for a given cell line when its logGI_50_ value was less than −8. We then assessed the actual experimental gene expression profiles of drug-treated cell lines using ssGSEA, and the predicted drug responses were in good agreement with the logGI_50_-based sensitivity classifications. For example, when NCI-H322M cells were treated with bortezomib and the logGI_50_ value exceeded the −8 threshold, the drug was classified as ineffective according to the ssGSEA-derived signatures of the six transcriptomic states. The results are shown in Figure 8.

### 2.6. Application of GRIP-Lung Integrated with ssGSEA for Drug Response Prediction

We further applied GRIP-Lung to predict drug-induced gene expression profiles in cell lines treated with compounds for which transcriptomic data are unavailable. Despite the absence of experimental data of 5-azacytidine treatment in the HCC515 cell line, our model reliably predicted the resulting gene expression profiles, demonstrating an additional application of GRIP-Lung in predicting drug-induced transcriptional changes in untested cell lines.

In addition, to evaluate the translational potential of GRIP-Lung in drug screening, we systematically applied a model to predict the gene expression responses of NSCLC patient samples under various drug perturbations. The gene expression data of the lung cancer patients used were obtained from TCGA-LUAD. The predicted gene expression profiles were subsequently analyzed using ssGSEA to assign cells to distinct post-treatment response states on the basis of selected sets of upregulated marker gene sets, highlighting its potential for drug repurposing and the discovery of novel therapeutic candidates. This classification strategy enabled the identification of transcriptionally and mechanistically relevant candidate drugs, highlighting the utility of GRIP-Lung as a generative screening tool for lung cancer drug repositioning.

Cancer is characterized by significant molecular, genetic, and phenotypic heterogeneity, and there is no single drug for cancer that can effectively treat all patients [31,32,33]. On the basis of these facts, treatment approaches should be personalized on the basis of the genomic characteristics of each patient. In accordance with this concept, our prediction results revealed that the treatment response state to erlotinib for each of the 585 TCGA-LUAD gene expression profiles was diverse, as indicated by the samples (Figure S5). This ssGSEA-guided classification improves the visualization of the intrinsic variation of tumor cells beyond that captured by a simple ON/OFF effectiveness status for a given drug. The combination of GRIP-Lung predictions and ssGSEA state assignments supports a mechanistically informed drug prioritization and repurposing strategy yielding hypotheses testable through biological interpretation prior to performing additional experiments. In addition, this approach facilitates the discovery of compounds that selectively modulate unique cellular programs, thereby guiding the design of more targeted therapeutic approaches against cancer.

## 3. Discussion

In this study, we constructed a drug response prediction model based on a GAN and its principles and established complete and comprehensive evaluation metric methods to test it. The results indicate that the drug response prediction models we developed have high prediction accuracy and strong stability. These results offer insight into the drug response patterns of lung cancer cells and support a new approach for identifying important genes and pathways related to drug response.

While providing accurate overall predictions, this model also provides biologically plausible evidence of the potential mechanisms governing the responses to different drugs. For instance, when erlotinib was used to treat lung cancer cell lines, our prediction model revealed that the genes affected by treatment were involved mainly in cell-cycle regulation. This has been documented by other studies in which the stimulation of such pathways in response to erlotinib inhibits cell proliferation and survival.

Case-specific interpretability suggests that GRIP-Lung can be used in more applications than merely for prediction; it can be further employed as an inference tool to contribute greatly to the study of drug mechanisms. Thus, GRIP-Lung improves clinicians’ judgment regarding therapy selection for lung cancer patients. The GRIP-Lung generator employs a residual network as its main feature extractor, allowing it to perform well with regard to predicting the effects on gene expression when tested using synthetic and real-world compound data. The integration of cell line and drug-specific information into the initial representations learned by the network allows the more adequate capture of sample-specific variance in biological responses. Traditional model approaches work on individual gene expression in isolation from the expression of other genes, whereas our approach explores their interconnectedness. This architecture has an embedded aux condition, which makes this neural network adaptable and interpretable. The ResNet-based encoder can model complex nonlinear dependencies between genes effectively, which is convenient for generating heterogeneous omics datasets, mainly without any a priori domain-specific knowledge about certain gene interactions or their combinations. Residual connections can solve the vanishing gradient problem such that the deep network can be trained more smoothly. These properties enable the ability of GRIP-Lung to capture complex transcriptional perturbation patterns, and in combination with a lightweight MLP discriminator, GRIP-Lung can achieve stable adversarial training to distinguish real from generated gene expression profiles for better prediction; thus, GRIP-Lung is a strong candidate tool for precision medicine, as the major point of inference of transcriptional response should be highly accurate no matter how the cells or drugs are transformed under different conditions.

On the basis of the ssGSEA framework, drug-induced transcriptional changes can be summarized into six key cellular states, allowing the functional comparison of varying heterogeneous samples while moving away from a binary approach of effective versus ineffective. Nevertheless, these states fail to entirely cover the entirety of cancer biology, as important processes such as metabolic reprogramming or EMT are lacking. In addition, several of these states contain overlapping pathways, leading to uncertainty in categorizing the data obtained from the network analysis process.

GRIP-Lung also provides a transcriptome-driven framework for computational drug repositioning by modeling drug-induced state transitions. By predicting post-treatment gene expression profiles from baseline states and quantifying functional program shifts using GSEA, the model enables the prioritization of compounds that drive tumor cells toward favorable biological states, such as enhanced programmed cell death or suppressed resistance pathways. Unlike approaches based primarily on chemical similarity or predefined targets, this strategy evaluates drugs according to their systems-level regulatory impact on cancer-associated gene programs. Although the present implementation is restricted to a limited number of lung cancer cell lines and compounds, the framework is readily extensible to larger pharmacogenomic datasets and broader drug libraries. At the current stage, the framework supports transcriptome-driven computational drug repositioning by prioritizing candidate compounds on the basis of predicted state transitions. It should be regarded as a hypothesis-generating tool that guides subsequent experimental and clinical validation rather than as definitive clinical repurposing evidence.

Although favorable performance was achieved during the evaluation of this model, several limitations still exist. First, the present model is insufficient for considering the importance of the tumor microenvironment in influencing drug responses, such as those involving infiltrating immune cells and their intercellular interactions, which can strongly impact the effectiveness of therapies. Importantly, GRIP-Lung was trained exclusively on cancer cell line RNA-seq data that lacked a complete TME context. Although moderate concordance was observed between the predicted transcriptional changes and patient-derived bulk RNA-seq profiles, bulk tumor samples inherently contain both malignant and microenvironmental components. Therefore, this concordance likely reflects the ability of the model to capture tumor cell-intrinsic transcriptional responses rather than TME-mediated effects. TME-associated processes, such as immune infiltration, stromal signaling, or hypoxia-driven transcriptional adaptation, are not explicitly modeled in the current framework and represent important limitations. In addition, drug dosages and duration of exposure were not incorporated into the model. These pharmacological parameters can significantly influence transcriptional outcomes and may contribute to variability in clinical drug response.

Another limitation concerns generalization across drugs and cellular contexts. GRIP-Lung relies on learned drug embeddings derived from compounds included in the training set; therefore, it cannot directly predict responses to entirely unseen drugs without retraining. Although the model may extrapolate to transcriptionally similar lung cancer cell lines within the training distribution, its performance in more divergent contexts remains uncertain. Future work could incorporate structure-based drug representations, such as SMILES-derived embeddings, to improve the generalizability to novel compounds, requiring expanded and more diverse training data. Furthermore, autophagy and senescence play dual roles in cancer. Protective autophagy may increase tumor survival under therapeutic stress, and the SASP can promote tumor progression. Thus, the interpretation of these states in our model should be understood within the framework of predicted transcriptional programs, rather than as definitive biological outcomes.

To overcome the aforementioned drawbacks, in the near future, a multi-modal model could be explored to utilize single-cell transcriptome information, spatial transcriptomics, and even multi-omics analysis to integrate more detailed intratumor heterogeneity and a comprehensive description of the TME. Moreover, widening the data scope, broadening the training datasets, and improving biological interpretation will facilitate the development of these types of models as robust solutions for creating powerful AI algorithms in precision oncology.

## 4. Materials and Methods

### 4.1. Data Collection and Preprocessing

Gene expression profiles of lung cancer cell lines, both untreated and treated with various anticancer drugs, were obtained from the publicly available Gene Expression Omnibus (GEO) database. Additional data were obtained from the LINCS L1000 dataset, which similarly includes the transcriptional profiles of untreated and drug-treated cells. Each profile contains gene expression data following treatment with drugs such as topotecan and erlotinib, among others. These data served as the input and output pairs for training the GAN model. The full list of drugs, corresponding GEO accession numbers, and associated cell lines is provided in Supplementary Table S4.

Gene expression data were obtained as processed files (series matrix or normalized expression values, when available) and log2-transformed. The baseline gene expression profiles correspond to pre-treatment expression measurements for lung cancer cell lines under untreated control conditions, as provided in the original datasets. Only lung cancer cell lines with both untreated (baseline) samples and corresponding drug-treated samples were retained for analysis. All the samples were transposed such that each row represented a gene and each column represented a sample. Standard normalization was performed using z score transformation for both the input (pre-treatment) and output (post-treatment) expression matrices. Specifically, the TPM values were transformed using log2 (TPM + 1) prior to normalization. For each dataset, z score normalization was applied independently to the input (pre-treatment) and output (post-treatment) expression matrices across samples for each gene. The normalization parameters were computed within each cross-validation training fold and then applied to the corresponding validation and test sets to avoid data leakage. The normalized data were subsequently converted into PyTorch tensors (v1.12.1; Meta AI, Menlo Park, CA, USA) for model training.

### 4.2. Model Architecture of GRIP-Lung

We propose a GAN-based approach for generating drug expression profiles. The GAN framework comprises two main components: a generator and a discriminator. The generator maps undrugged expression profiles to drugged expression profiles by minimizing reconstruction error, with mean squared error (MSE) as the loss function. As the generator feeds its output into the discriminator, the task of the latter is to judge whether a certain profile originates from the drug expression distribution to provide feedback on how to adjust the model to further reduce the error rate. The input and output file formats used in this model are publicly available in our GitHub repository to facilitate reproducibility.

### 4.3. Generator and Discriminator Combinations Tested

The model was built upon the GAN framework, comprising multiple generator and discriminator architectures. Three generator variants, namely, a conditional autoencoder (cAE), a transformer, and a residual network, and two discriminator variants, namely, a 1D convolutional neural network (1D-CNN) and a lightweight multilayer perceptron (MLP), were designed, resulting in six possible GAN configurations. These configurations are summarized in Table 2. The configuration that achieved the best performance was selected as the final model.

Among the six evaluated architectures (cAE-MLP, cAE-1D-CNN, Transformer–MLP, Transformer–1D-CNN, Residual–MLP, and Residual–1D-CNN), Residual–MLP demonstrated the best performance. Therefore, we provide a detailed description of the Residual generator and MLP discriminator below. The configurations of the other architectures are reported in Supplementary Note S1.

### 4.4. Embedding of Cell Lines and Drugs

To integrate contextual biological information into the model, we applied an embedding strategy for both cell lines and drug identifiers. Each embedding vector was trained to capture latent characteristics of the corresponding entity and was projected to match the dimensionality of the input gene expression profile. The cell line and drug embeddings were then added element-wise to the normalized input expression vector, enabling the generator to learn drug- and cell-specific perturbation patterns during training. This element-wise integration avoided increasing the input dimensionality, as would occur with simple concatenation, and ensured efficient conditioning while maintaining the interpretability of gene-level features. Each embedding vector had a dimensionality of 128, a choice that provided sufficient capacity to capture latent biological and pharmacological features while maintaining a balanced contribution to the hidden representation of the generator.

### 4.5. Residual Network Generator

The generator was designed as a residual conditional network, where condition information is incorporated by embedding layers to predict drug-induced transcriptional perturbations. The input to the generator consists of the baseline gene expression profile *x* along with the identifiers of the cell line and the administered drug. The cell lines and drug identities are first embedded into low-dimensional vectors, which are concatenated and passed through a modulation network to generate two conditioning parameters, *γ* and *β.* These parameters are applied to the hidden features via feature-wise linear modulation (FiLM), enabling the network to adaptively adjust its feature activations according to the specific cell–drug combination.

A residual block is then applied to facilitate gradient propagation and stabilize training. The generator outputs a residual vector Δ*x*, representing the predicted transcriptional shift induced by drug treatment, which is added to the original baseline profile to produce the final predicted expression:

(1) $$\tilde{y}=x+\Delta x=x+G(x,cell,drug)$$

This residual formulation emphasizes learning relative rather than absolute differences, which leads to more stable convergence and a more interpretable biological space. In addition, the generator is trained using a hybrid loss that combines an adversarial term with a regression constraint to produce both realistic gene-level prediction distributions and high numerical accuracy.

Specifically, the generator projects the input gene expression vector to a hidden dimension of 256 using a fully connected layer. The subsequent residual block consists of two fully connected layers, each with 256 hidden units and ReLU activation functions. The FiLM-style modulation parameters, *γ* and *β*, are applied after this projection to condition the hidden features on the cell line and drug embeddings. Finally, a linear layer maps the processed hidden representation back to the original gene dimension, and the resulting residual vector is added to the baseline expression to form the predicted transcriptional response. This detailed architecture allows the network to perform deep feature transformations while preserving direct gene-level correspondence, facilitating stable training and accurate perturbation prediction.

### 4.6. Lightweight MLP Discriminator

The discriminator was formulated as a lightweight multilayer perceptron for distinguishing gene expression profiles on the basis of whether they were derived from real or fake samples under a given condition. Each input consisted of a gene expression vector together with the embeddings of the corresponding cell type and drug.

The network consists of three fully connected layers with LeakyReLU activation functions and one output neuron, yielding a scalar value representing whether the input data corresponded to experimental measurements of drug cell expression or was synthesized using our generative model. This simple design allows the number of parameters to remain low, thereby reducing the risk of overfitting and making training stable. Despite its simplicity, the discriminator manages to identify correlations in drug cell expression and gives robust adversarial signals to the generator.

### 4.7. Adversarial Training Strategy

The conditional GAN was trained using an adversarial process in which the aim of the generator is to make the predicted post-treatment transcriptional profiles minimally different from the real ones, and the task of the discriminator is to maximize the discrepancy between them. Additionally, the generator employs an adversarial strategy coupled with a regression constraint on the noise term, ensuring that the output is not only plausible but also numerically close to the expected values. The specific definitions of the loss function terms are shown below:

(2) $$L_{G}=\mathrm{MSE}(G(x),y)+\lambda \cdot E_{x\sim P_{\mathrm{data}}}\left[-\log(D(G(x)))\right]$$

(3) $$L_{D}=-E_{y\sim P_{\mathrm{data}}}\left[\log D(y)\right]-E_{x\sim P_{\mathrm{data}}}\left[\log(1-D(G(x)))\right]$$

where *λ* is a weighting hyperparameter that balances the adversarial and regression objectives.

The adversarial term encourages the generator to produce realistic drug response distributions, whereas the MSE term constrains the output to remain numerically consistent with ground-truth post-treatment gene expression.

### 4.8. Training Procedure

The model was trained using the Adam optimizer with a learning rate of 0.001. During each epoch, the discriminator and generator were alternately updated: the discriminator was first optimized to distinguish real from generated samples, and the generator was then optimized to both fool the discriminator and minimize reconstruction error. The MSE between the predicted and actual expression profiles was used to monitor the regression accuracy of the generator output.

### 4.9. Extension to Patient-Derived Transcriptomic Data

To evaluate the translational potential of our model, we applied a trained generator that was originally developed on lung cancer cell line data to bulk RNA-seq data derived from lung cancer patients. A publicly available dataset (GSE165019) was used as an external source for patient transcriptome profiles. Four samples whose clinical annotations included erlotinib treatment information were selected for analysis.

Patient gene expression data were preprocessed to match the input format of the generator, including gene filtering and normalization procedures identical to those applied to the training data. The untreated expression profile of the patient was mapped to the most similar cell line in the training dataset on the basis of expression similarity, and the corresponding cell embedding was used for prediction, while the drug embeddings were maintained as in the original model. To evaluate the robustness of the model, we compared the predicted expression profiles of post-treatment patients with their corresponding actual profiles. Expression correlation metrics were used to evaluate the consistency between the predicted responses and observed patterns.

### 4.10. ssGSEA-Based Approach for Identifying Drug Post-Treatment States

To study the biological effects of the post-treatment predicted expression profile, we constructed an encompassing set of representative biomarkers that capture various major cellular states we tested, such as programmed cell death (apoptosis, autophagy, and ferroptosis), senescence, cell-cycle arrest, drug resistance, immune escape, and malignant progression. The upregulated expression of all selected biomarkers was used as an indicator of the aforementioned cellular states. Notably, certain biological processes, such as autophagy and senescence, are highly context-dependent. In the present study, the upregulation of the expression of biomarkers was operationally interpreted as reflecting treatment-induced stress and growth suppression in vitro, rather than long-term tumor-promoting effects such as senescence-associated secretory phenotype (SASP)-mediated progression. We used the single-sample gene set enrichment analysis (ssGSEA) scoring method to classify predicted expression profiles on the basis of each biomarker signature to determine whether treatment-induced expression patterns, such as gene programs for programmed cell death, cell-cycle arrest, and senescence, represented an effective therapeutic response or states associated with ineffective therapy, such as drug resistance, immune escape, and tumor progression. The details of the gene sets representing different post-treatment response states are provided in Supplementary Table S5. We evaluated the performance of ssGSEA using the logGI_50_ from the NCI-60 cell line panel as a measure of drug sensitivity and calculated effective scores as mean z scores from the effective gene sets [34]. Ineffective scores for the ineffective gene sets were computed similarly, and the difference from the effective score was used to calculate the resistance index (RI) for every sample, providing an indication of whether the associated pathway was relatively more activated. If a sample had an RI value greater than zero, then the sample was regarded as ineffective; otherwise, if an RI value was equal to or less than zero, the sample was considered to be effective, thus also representing the opposite sides of transcriptional program activity following drug treatment.

## 5. Conclusions

To conclude, we put forward the proposed GAN model that can predict drug-induced gene expression alterations for different lung cancer cells. Utilizing biologically meaningful embeddings greatly increases performance in both high-precision and low-precision learning, while providing in-depth insights into biological mechanisms to help us understand the mechanisms of drug response. This framework establishes a foundation for developing more personalized and mechanism-driven therapeutic strategies, thereby advancing the broader goals of data-driven precision medicine.

## Figures and Tables

**Table 1:** Performance comparison of different algorithms based on various evaluation metrics.

Combination	MSE	R^2^	ACC	Prec	Rec	F1-Score
cAE + MLP	0.747 ± 0.097	0.246 ± 0.087	0.695 ± 0.021	0.695 ± 0.02	0.694 ± 0.02	0.694 ± 0.021
cAE + CNN	1.04 ± 0.081	−0.052 ± 0.054	0.567 ± 0.038	0.567 ± 0.038	0.566 ± 0.037	0.564 ± 0.037
Res + MLP	0.337 ± 0.022	0.652 ± 0.036	0.84 ± 0.008	0.839 ± 0.008	0.839 ± 0.008	0.839 ± 0.008
Res + CNN	0.346 ± 0.020	0.643 ± 0.034	0.835 ± 0.008	0.835 ± 0.008	0.835 ± 0.008	0.835 ± 0.008
Trans + MLP	1.627 ± 0.251	−0.663 ± 0.269	0.513 ± 0.023	0.515 ± 0.026	0.515 ± 0.025	0.507 ± 0.026
Trans + CNN	2.629 ± 1.186	−1.714 ± 1.236	0.501 ± 0.001	0.5 ± 0.002	0.5 ± 0.002	0.497 ± 0.005

**Table 2:** Tested generator–discriminator combinations in the GAN framework.

Generator	Discriminator	Combination
cAE	MLP	cAE-MLP
cAE	1D-CNN	cAE-1D-CNN
Transformer	MLP	Transformer–MLP
Transformer	1D-CNN	Transformer–1D-CNN
Residual network	MLP	Residual–MLP
Residual network	1D-CNN	Residual–1D-CNN

## Data Availability

The data presented in this study are openly available in the TCGA-LUAD database at https://portal.gdc.cancer.gov/ and the Gene Expression Omnibus (GEO) at https://www.ncbi.nlm.nih.gov/geo/ (both accessed 1 April 2026), with GEO reference numbers GSE92742, GSE116437, and GSE116451.

## Supplementary Materials

The following supporting information can be downloaded at https://www.mdpi.com/article/10.3390/ijms27073264/s1.

## Abbreviations

The following abbreviations are used in this manuscript:

GAN	Generative Adversarial Network
FiLM	Feature-wise Linear Modulation
DEGs	Differentially expressed genes
