# Supplementary material for: GRIP-Lung: Generative Model of Response to Drug-Induced Perturbation in Lung Cancer
Source: Int J Mol Sci. 2026 Apr 3;27(7):3264. doi: 10.3390/ijms27073264 (PMC13072768; doi:10.3390/ijms27073264)
Supplement: Supplementary file 1 [file ijms-27-03264-s001.zip › Supplementary Table S5.pdf]

Supplementary Table S5. The sets of biomarkers for six post-treatment response states.

Post-treatment response state	Biomarker
Programmed cell death (apoptosis & autophagy & ferroptosis)	*BAX, BAK, CYCS, DIABLO, BBC3, PMAIP1, MAP1LC3B, BECN1, ATG5, ATG7, BNIP3, WIPI2, ACSL4, PTGS2, CHAC1, TFRC, NCOA4,* and *ALOX15*
Cell cycle arrest	*CDKN1A, CDKN1B, GADD45A, WEE1, RB1, E2F7, E2F8, CDKN2A, SESN2, PRKAA1, PRKAA2, MAPK14, RBL2,* and *E2F4*
Senescence	*CDKN2A, CDKN1A, TP53, GLB1, IL6, CXCL8, MMP1, MMP3, CCL2, SERPINE1, GDF15, IGFBP7, IGFBP3, H2AFY, CXCL1, CXCL2,* and *TIMP1*
Drug resistance	*ABCB1, ABCG2, ABCC1, ERCC1, ZEB1, SNAI1, VIM, ALDH1A1, CD44, SOX2, MCL1, BCL2, CD274, FGFR1,* and *NFE2L2*
Immune escape	*CD274, PDCD1LG2, IDO1, CD47, HLA-G, CD276, LGALS9, CEACAM1, ADAM10, ADAM17, IL10, NT5E, HLA-E,* and *FGL1*
Malignant progression	*MKI67, CCND1, FOXM1, MMP9, VIM, ZEB1, SNAI1, TWIST1, AXL, MET, VEGFA, HIF1A, CXCR4, CD44, ALDH1A1, SOX2, TERT, CD274, S100A4,* and *RELA*
