# Supplementary material for: GRIP-Lung: Generative Model of Response to Drug-Induced Perturbation in Lung Cancer
Source: Int J Mol Sci. 2026 Apr 3;27(7):3264. doi: 10.3390/ijms27073264 (PMC13072768; doi:10.3390/ijms27073264)
Supplement: Supplementary file 1 [file ijms-27-03264-s001.zip › Supplementary Note S1.pdf]

Conditional AutoEncoder Generator

The Conditional AutoEncoder (cAE) Generator was designed to learn the conditional transformation from pre-treatment to post-treatment gene expression profiles by incorporating contextual information from both cell line and drug identities. The architecture follows the general principle of an autoencoder but is extended with conditional inputs to guide the encoding–decoding process.

Specifically, the generator receives a concatenated input vector composed of the normalized pre-treatment gene expression vector $x \in \mathbb{R}^G$, together with the encoded representations of drug (*d*) and cell line (*c*) conditions. These conditional variables are numerically encoded and appended to the gene expression vector, forming the joint input [*x*, *d*, *c*]. The model architecture consists of two main components: an encoder and a decoder.

The encoder compresses the concatenated input into a lower-dimensional latent representation through two fully connected layers with ReLU activation functions:

$$h = \mathrm{ReLU}\left(W_2\,\mathrm{ReLU}\left(W_1[x,d,c]+b_1\right)+b_2\right)$$

where W_1, W_2 and b_1, b_2 denote the learnable parameters.

The decoder subsequently reconstructs the post-treatment gene expression profile ŷ from the latent representation:

$$\hat{y} = W_4\,\mathrm{ReLU}\left(W_3 h + b_3\right)+b_4$$

The network is trained to minimize the mean squared error (MSE) between the predicted and the true post-treatment gene expression vectors, while optionally participating in the adversarial optimization process of the GAN framework to improve

realism in generated profiles.

In this setup, the inclusion of the drug and cell line variables as conditional factors enables the generator to produce gene expression responses that are context-specific, thereby modeling the pharmacogenomic interaction between compounds and cellular systems. This design ensures that the learned mapping reflects biologically meaningful treatment-specific transcriptional patterns.

Transformer Generator

The Transformer Generator is designed to capture complex dependencies among gene features while incorporating the same conditional information. It takes the concatenated input vector $\tilde{x} = [x, d, c]$ as defined above.

The input vector is first projected into a latent representation via a fully connected layer:

$$h_0 = W_{\text{input}}\,\tilde{x} + b_{\text{input}}$$

The latent representation is then processed by a Transformer encoder:

$$H = TransformerEncoder\left(h_0\right)$$

Finally, the output is mapped back to the gene expression space:

$$\hat{y} = W_{\text{output}}\,H + b_{\text{output}}$$

The generator is trained with mean squared error loss and optionally adversarial loss within the GAN framework. The conditional embeddings ensure that the generated gene expression profiles are treatment- and cell line-specific.

1D-CNN Discriminator

The 1D-CNN Discriminator distinguishes between real post-treatment gene expression profiles and those generated by the generator.

The discriminator receives the post-treatment gene expression vector, denoted as x_{gene}, and the conditional embeddings d and c. The gene expression vector is reshaped as a one-dimensional sequence and processed through a stack of 1D convolutional layers with LeakyReLU activation:

The flattened convolutional features are then concatenated with the condition embeddings, and a fully connected layer produces the final classification logit:

$$h_1 = \mathrm{LeakyReLU}\left(\mathrm{Conv1D}_1\left(x_{\mathrm{gene}}\right)\right)$$

$$h_2 = \mathrm{LeakyReLU}\left(\mathrm{Conv1D}_2\left(h_1\right)\right)$$

The discriminator is trained using binary cross-entropy loss to correctly classify real versus generated profiles, providing adversarial feedback to improve the generator's output realism.

$$\mathrm{logit} = W_{\mathrm{fc}}\left[h_2 \| d, c\right] + b_{\mathrm{fc}}$$
