# Supplementary material for: GRIP-Lung: Generative Model of Response to Drug-Induced Perturbation in Lung Cancer
Source: Int J Mol Sci. 2026 Apr 3;27(7):3264. doi: 10.3390/ijms27073264 (PMC13072768; doi:10.3390/ijms27073264)
Supplement: Supplementary file 1 [file ijms-27-03264-s001.zip › Supplementary Table S1.pdf]

Supplementary Table S1. Comparison of predicted and actual up- and down-regulated DEGs (top 100).

	Genes
Predicted down-regulated DEGs	*FOSL1, FJX1, E2F8, HMGA2, LMNB1, AMOTL2, FBXO5, LIF, TFAP2A, CCNF, PLK1, CCL2, PHLDA1, PPRC1, RRS1, RIN2, SPRY2, TSEN2, CENPA, IER3, SKP2, PSRC1, CDC25A, HJURP, MCM10, DUSP7, EREG, ERCC6L, GTSE1, MCM7, CLCF1, MAP3K14, POP1, ALDH1A3, BARD1, CDCA4, SUV39H2, ARHGAP19, GINS1, LANCL2, KIF15, DBF4, MCM4, AURKB, POLE2, WEE1, ZC3H4, BIRC5, GEMIN6, BNC2, RRM2, WT1, MLPH, AGO2, SRSF6, NOP16, SH3BP4, ARHGAP11A, DEPDC1, CDC7, TNS3, MOK, COIL, SERTAD3, ATAD2, CDCA3, RGS20, PLK4, FERMT1, SUV39H1, F2RL1, MKI67, ZNF573, CCNA2, HSPA14, MSL1, GINS3, NFKB1, FOXF2, KIF11, SRSF4, CHD7, PAGR1, DUSP4, AURKA, MAT2A, ZBED5, EXOSC4, METTL1, RBL1, TDP1, EED, EPHA2, MCM5, SCO2, EXO1, ERF, MEPCE, TMA16,* and *ZNF587B*
Predicted up-regulated DEGs	*DDIT4, DDIT3, ATF3, CHAC1, HSPA6, ASNS, PCK2, PHGDH, CTH, HSPB8, HERPUD1, TNFSF9, CCNG2, SAT1, PGF, TRIB3, LARP6, CDKN1A, ARL4D, BCL6, PSAT1, DNAJB9, ASS1, PNRC1, NFIL3, XBP1, DGKA, NEU1, DNAJB4, TSPAN7, ANKRA2, BAMBI, GADD45A, STC1, NEAT1, BTG1, AHNAK2, TRIM2, SPP1, ERBB3, IDH1, RNASE4, TPP1, NTS, TUBB2B, SERPINB1, ULBP2, CLGN, APOBEC3G, SLC2A3, WIPI1, FOS, NDRG4, BEX4, IFI16, ALDH1A1, MSX1, DNAJC6, NUPR1, MT1F, NDRG1, DNAJC12, ISG15, SLC1A4, IFIT1, SERPINI1, CHMP1B, BNIP3L, GRB10, HS1BP3, GOT1, CEBPB, MKNK2, TP53TG1, SCPEP1, DNAJB1, GABARAPL1, DAAM1, HEY1, PHYH, CSRNP2, CLK1, BCHE, CD55, C14orf132, TXNIP, MBNL2, FAM117A, TRIM36, HSPA2, PMAIP1, PRPH, RAB33B, KCNJ2, MICB, GLRX, ISG20, RIOK3, TNFRSF9,* and *P4HA2*
Actual down-regulated DEGs	*PSRC1, PLK1, LMNB1, FJX1, FOSL1, CENPA, CCNA2, HMGA2, TNS3, HJURP, CCNF, BORA, GTSE1, LIF, LRP8, CDCA3, ARHGAP11A, DEPDC1, SKP2, KIF20A, AURKA, CDCA8, KIF15, GEMIN4, ARHGAP19, KIF23, FBXO5, TSEN2, PLK4, CDC20, SPC25, KIF18B, CENPE, CCND1, MALL, E2F5, CTPS1, MKI67, NAV3, BUB1B, NRF1, RCC1, DBF4, RRS1, NDC80, URB2, POLE2, NCAPH, CDCA4, E2F8, CUTC, ERCC6L, MEIS2, FOXM1, HEATR3, KIFC1, GINS1, SRSF6, FXN, MAT2A, TDP1, CCNB1, HDAC4, PAGR1, PPRC1, RASA1, PHLDA1, UTP20, RGS4, UCK2, WDR43, ECT2, POLR3G, CDC25A, SLC25A12, NREP, ROR1, RIN2, KIF11, PFAS, SUV39H1, NSMAF, LMNB2, AURKB, TIPIN, FOXD1, ASPM, VANGL1, TLK1, CDC7, NEIL3, CENPI, RRP15, STK39, SMAD7, STAG1, BCAR3, TACC3, WDR76,* and *RGS20*
Actual up-regulated DEGs	*ATF3, DDIT3, FOS, CCNG2, DNAJB9, CHAC1, DUSP1, TNFSF9, BTG1, CTH, RHOB, NEU1, PGF, PNRC1, GABARAPL1, HERPUD1, ULBP2, TXNIP, PMAIP1, HSPA6, ASNS, BAMBI, GADD45B, CITED2, SAT1, NEAT1, YPEL5, PELI1, PCK2, MAFB, NRIP3, CDKN1A, ANKRA2, PPP1R3C, CLK1, NDRG1, HECA, BCL6, HLA-E, DDIT4, GADD45A, MAP1LC3B, TUBB2B, ARC, HSPB8, NFIL3, ZNF222, CXCL8, TRIM36, SERPINI1, WIPI1, LIN37, PPP1R15A, CSTA, RGS2, CLGN, DNAJB4, PI4K2A, MICB, CD55, TSPYL2, NR4A2, AVPI1, CLU, IFIT1, CHMP1B, PTGS2, TRIB3, ISG15, ARID5B, CSRP2, FAM53C, KLF5, TUFT1, RIOK3, RWDD2A, ABCA5, WDR47, GCH1, GPX3, GRN, CSRNP2, STX5, KDM7A, MOAP1, LARP6, GSTM3, GTF2B, ARL4D, TOB1, CYFIP2, LGMN, CEBPB, ZNF394, DUSP5, LBH, RSRP1, SCPEP1, PSAT1,* and *ZNF419*
