# Supplementary material for: GRIP-Lung: Generative Model of Response to Drug-Induced Perturbation in Lung Cancer
Source: Int J Mol Sci. 2026 Apr 3;27(7):3264. doi: 10.3390/ijms27073264 (PMC13072768; doi:10.3390/ijms27073264)
Supplement: Supplementary file 1 [file ijms-27-03264-s001.zip › Supplementary Table S2.pdf]

Supplementary Table S2. DEGs identified from true post-treatment versus baseline patient samples.

Gene	logFC	P.Value	Regulation
APOE	2.207	0.001	up
C3	1.545	0.009	up
RPS10	2.063	0.042	up
MMP9	1.914	0.006	up
UBTD1	2.028	0.014	up
PKIG	1.623	0.05	up
CEBPA	1.663	0.003	up
IFIT1	1.832	0.02	up
ADH1B	2.747	0.002	up
USP18	1.719	0.021	up
RBP4	2.08	0.001	up
KLF15	1.517	0.031	up
TBX3	1.716	0.044	up
ICAM3	1.708	0.011	up
BCHE	1.919	0.027	up
IGF1	1.621	0.034	up
ACSM5	1.955	0.008	up
POSTN	-2.123	0.036	down
GLA	-2.039	0.047	down
BCL10	-2.297	0.021	down
PLAT	-2.304	0.023	down
CD58	-2.09	0.032	down
SRPX2	-2.019	0.015	down
EGLN3	-2.825	0.001	down
SYTL2	-2.216	0.016	down
ITGB8	-2.263	0.008	down
