# Supplementary material for: GRIP-Lung: Generative Model of Response to Drug-Induced Perturbation in Lung Cancer
Source: Int J Mol Sci. 2026 Apr 3;27(7):3264. doi: 10.3390/ijms27073264 (PMC13072768; doi:10.3390/ijms27073264)
Supplement: Supplementary file 1 [file ijms-27-03264-s001.zip › Supplementary Table S3.pdf]

Supplementary Table S3. RMSE and MAE calculated on DEGs for each patient sample to evaluate magnitude prediction accuracy of GRIP-Lung.

Sample	Pearson	MAE	RMSE
Patient.2	0.64	1.29	1.71
Patient.4	0.41	2.25	2.82
Patient.5	0.63	1.23	1.68
Patient.6	0.31	2.08	2.56
