# Supplementary material for: GRIP-Lung: Generative Model of Response to Drug-Induced Perturbation in Lung Cancer
Source: Int J Mol Sci. 2026 Apr 3;27(7):3264. doi: 10.3390/ijms27073264 (PMC13072768; doi:10.3390/ijms27073264)
Supplement: Supplementary file 1 [file ijms-27-03264-s001.zip › Supplementary Table S4.pdf]

Supplementary Table S4. Data sources and sample counts for each drug–cell line pair.

Drug	A549	HOP-62	HOP-92	NCI-H23	NCI-H322M	NCI-H460	HCC515*	Data source
5-Azacytidine	6	3	6	6	6	1	0	GSE116437
Bortezomib	6	6	6	6	6	6	37	GSE116438
Cisplatin	6	6	6	6	6	6	27	GSE116439
Dasatinib	6	6	6	6	4	6	3	GSE116440
Doxorubicin	6	6	6	6	6	6	30	GSE116441
Erlotinib	6	6	6	6	6	6	3	GSE116442
Geldanamycin	6	6	6	6	6	6	117	GSE116443
Gemcitabine	6	6	6	6	6	6	36	GSE116444
Lapatinib	6	6	6	4	6	6	6	GSE116445
Paclitaxel	6	6	6	6	6	6	38	GSE116446
Sirolimus	6	6	6	6	6	6	80	GSE116447
Sorafenib	6	6	6	6	6	6	6	GSE116448
Sunitinib	6	6	5	6	6	3	3	GSE116449
Topotecan	5	6	6	6	6	6	3	GSE116450
Vorinostat	6	6	6	6	6	6	120	GSE116451

*** Expression data for HCC515 cell line from LINCS (GSE92742, Level 3).**
