# Supplementary figures and images for: GRIP-Lung: Generative Model of Response to Drug-Induced Perturbation in Lung Cancer
Source: Int J Mol Sci. 2026 Apr 3;27(7):3264. doi: 10.3390/ijms27073264 (PMC13072768; doi:10.3390/ijms27073264)

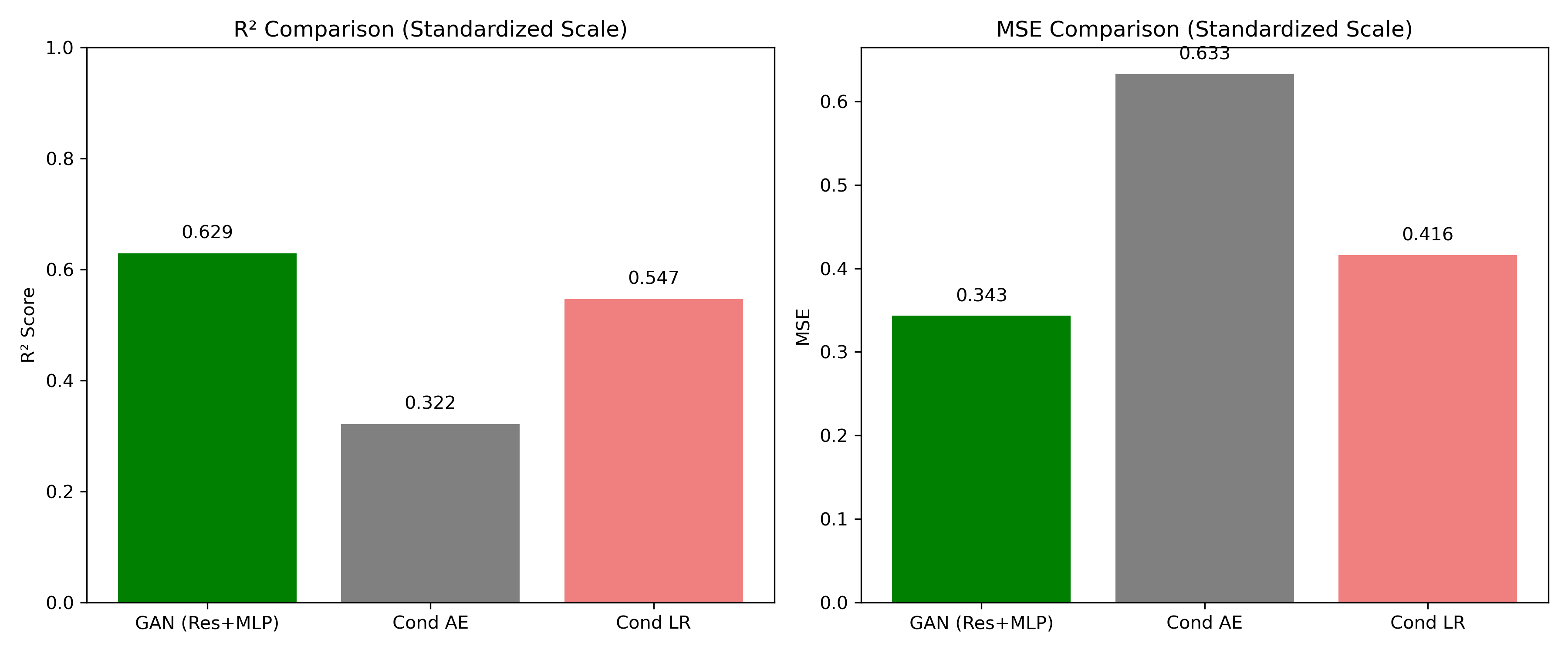

Supplement: Supplementary file 1 [file ijms-27-03264-s001.zip › Supplementary Figure S1.tif]

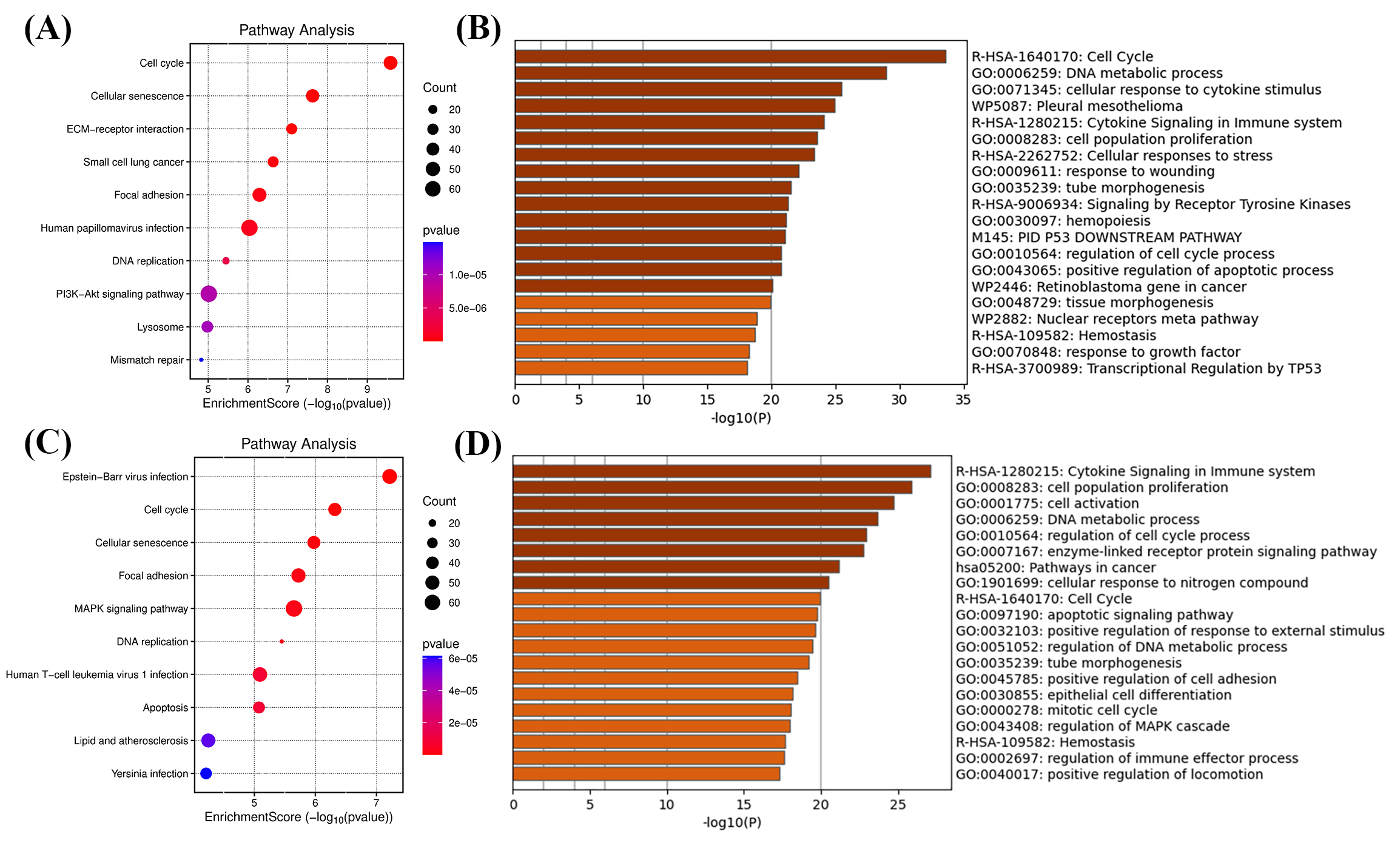

Supplement: Supplementary file 1 [file ijms-27-03264-s001.zip › Supplementary Figure S2.tif]

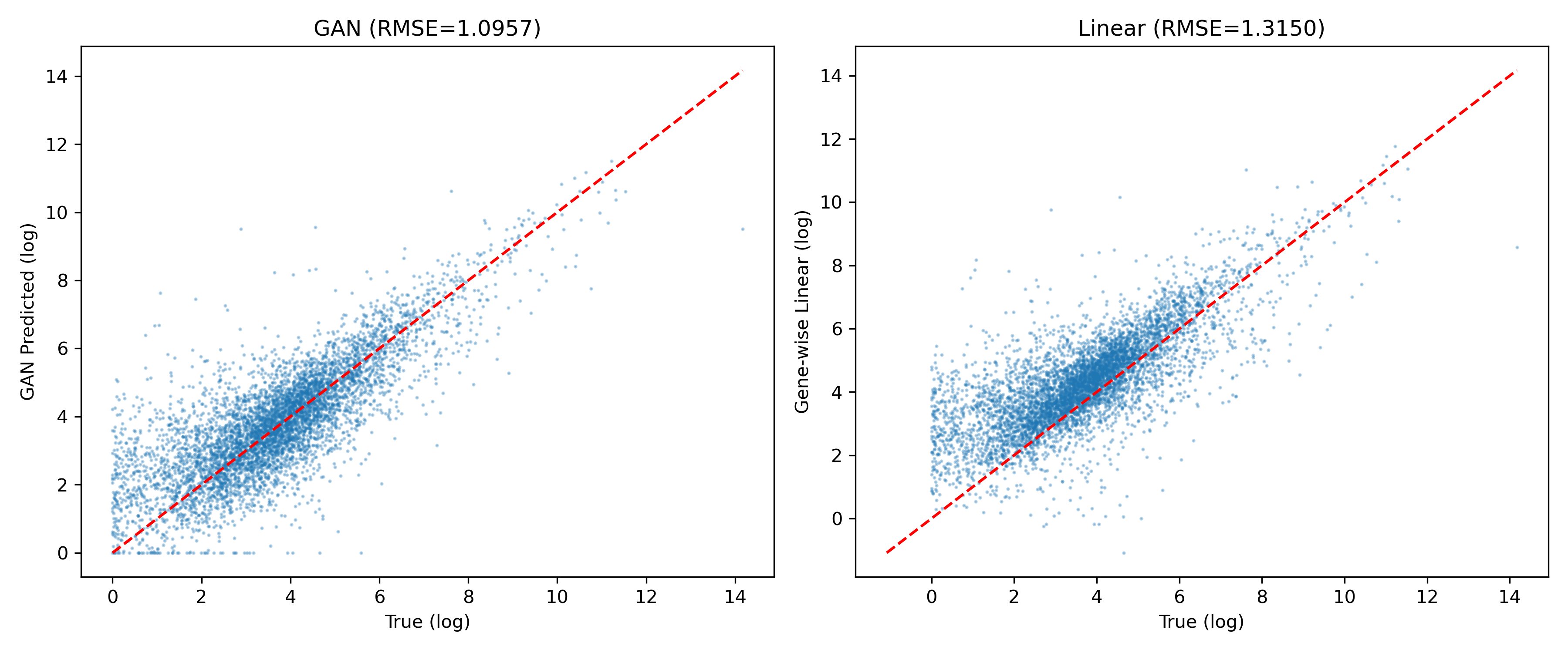

Supplement: Supplementary file 1 [file ijms-27-03264-s001.zip › Supplementary Figure S3.tif]

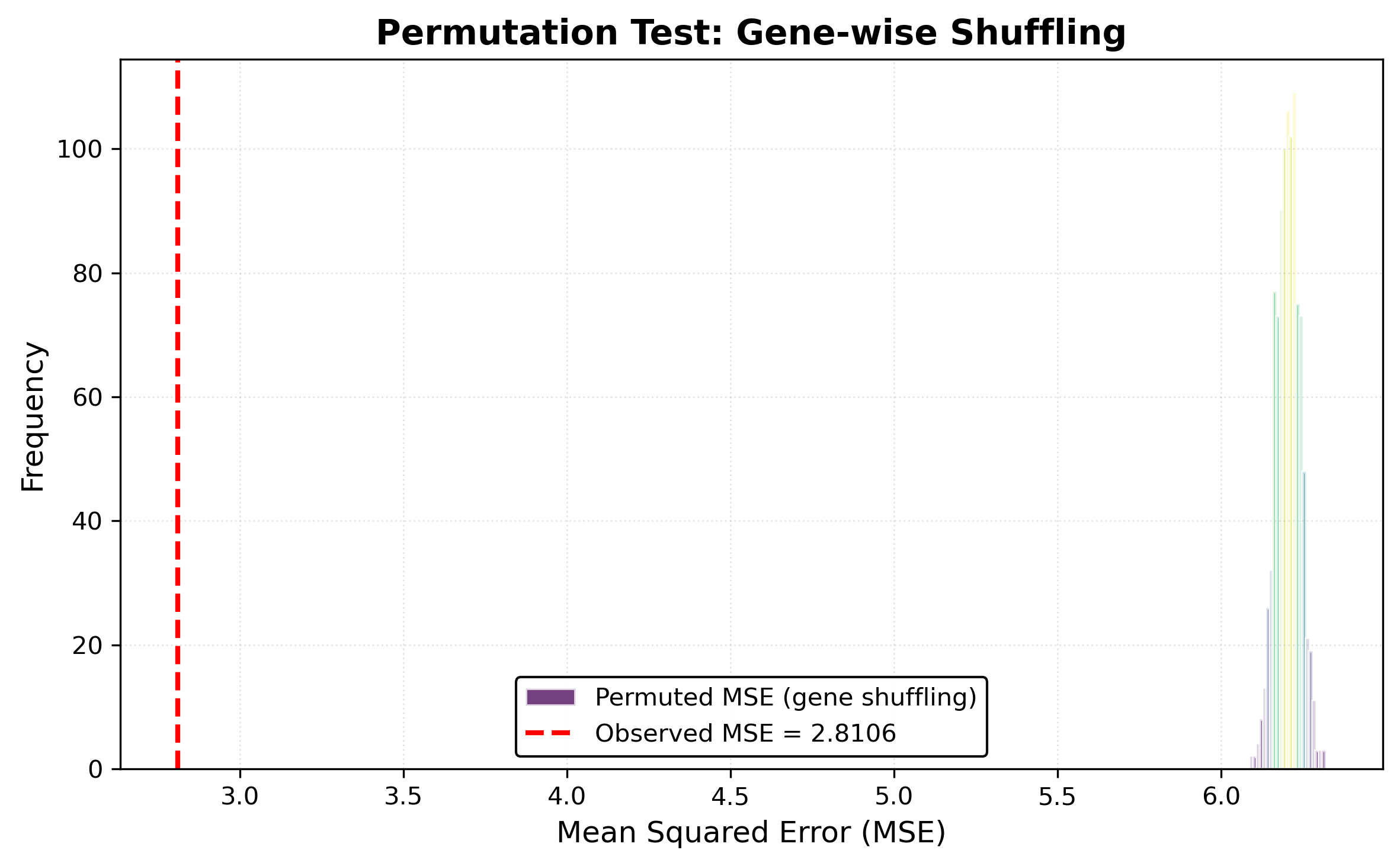

Supplement: Supplementary file 1 [file ijms-27-03264-s001.zip › Supplementary Figure S4.tif]

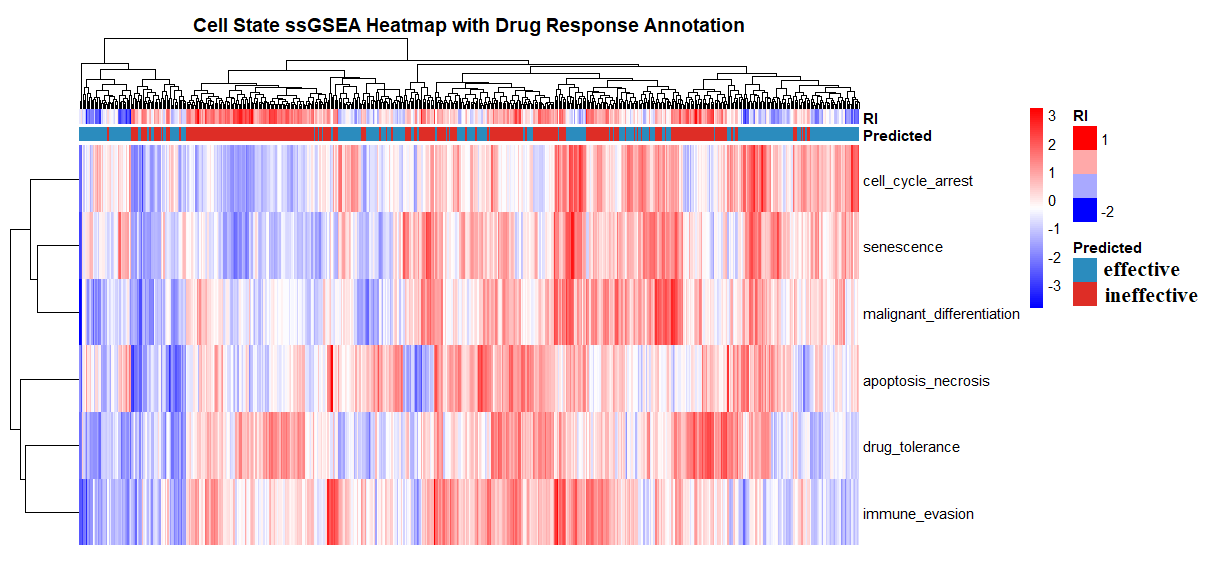

Supplement: Supplementary file 1 [file ijms-27-03264-s001.zip › Supplementary Figure S5.tif]
